# Fatal Case of Crimean-Congo Hemorrhagic Fever, Portugal, 2024

**DOI:** 10.3201/eid3101.241264

**Published:** 2025-01

**Authors:** Líbia Zé-Zé, Cristina Nunes, Micaela Sousa, Rita de Sousa, Carla Gomes, Ana S. Santos, Rui T. Alexandre, Fátima Amaro, Tiago Loza, Miriam Blanco, Maria J. Alves

**Affiliations:** National Institute of Health Doutor Ricardo Jorge, Águas de Moura, Portugal (L. Zé-Zé, R. de Sousa, A.S. Santos, F. Amaro, M.J. Alves); Center for the Study of Animal Science, Porto, Portugal (L. Zé-Zé, F. Amaro, M.J. Alves); Northeastern Local Health Unit, Bragança, Portugal (C. Nunes, M. Sousa, C. Gomes, R.T. Alexandre, T. Loza, M. Blanco); Environmental Health Institute, Lisboa, Portugal (A.S. Santos, F. Amaro, M.J. Alves)

**Keywords:** Crimean-Congo hemorrhagic fever, viral hemorrhagic fevers, vector-borne infections, tick-borne diseases, arbovirus, viruses, Crimean-Congo hemorrhagic fever virus, Portugal

## Abstract

We report a fatal case of Crimean-Congo hemorrhagic fever in Portugal. An 83-year-old man, initially suspected of having Mediterranean spotted fever, was later confirmed to have Crimean-Congo hemorrhagic fever by the detection of viral genome in the patient's serum and the presence of specific IgM antibodies.

Crimean-Congo hemorrhagic fever (CCHF) is a potentially severe or fatal disease caused by CCHF virus (CCHFV; species *Orthonairovirus haemorrhagiae*), a tickborne virus of the family *Nairoviridae*, order *Bunyavirales*. CCHF has a broad geographic distribution that includes Africa, Asia, the Middle East, and some eastern and southern European countries (*1*). In Spain, the first cases were reported in 2016 in the province of Ávila, Castilla-León region (*2*). To date, a total of 17 confirmed cases have been documented in Spain, including 4 cases reported in 2024; the province of Salamanca reported 1 case in April, Toledo reported 1 case in July, Córdoba (unconfirmed but most likely) reported 1 case in July, and Cáceres reported 1 case in August (*3*). In Portugal, antibodies to CCHFV were first detected in 2 human serum samples in 1985. Of the 2 patients, 1 showed clinical signs and symptoms compatible with CCHF (*4*). Since 1985, CCHFV has not been detected in ticks or humans, although several studies have been conducted on ticks. Beginning in 2020, surveillance has been conducted within the national vector surveillance network, which systematically collects ticks throughout the country (*5*).

CCHFV is maintained by both vertical and horizontal transmission cycles involving ticks, mostly of the genus *Hyalomma*, and various species of wild and domestic mammals and birds that develop transient viremia without showing signs of disease (*6*). Human infection occurs through tick bites (or exposure to arthropod fluids) or direct contact with the secretions, fluids, or tissues of viremic animals and humans, especially in the absence of appropriate protective measures (*7*,*8*). Although nosocomial infections and outbreaks have been reported, the risk of acquiring CCHFV from exposure to infected biologic fluids appears to be lower than for other hemorrhagic viruses, such as Ebola (*9*). Asymptomatic CCHFV infections are thought to be common and may account for up to 90% of cases in hyperendemic areas (*10*). However, CCHFV can cause a severe or fatal clinical course; mortality rates have ranged from 3% to 40% (*6*,*11*). In this article, we report a clinically documented case of autochthonous CCHF in Portugal with a fatal outcome.

## The Case

**Figure 1:**
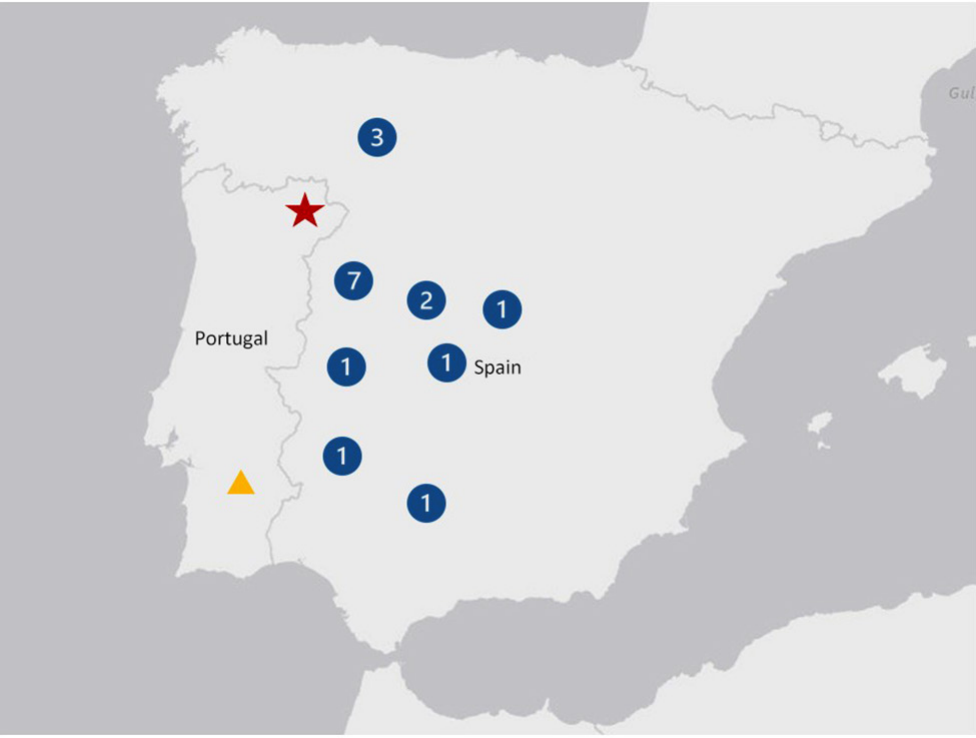
Regions of the Iberian Peninsula where human infections with Crimean-Congo hemorrhagic fever virus were reported. Red star indicates fatal case of Crimean-Congo hemorrhagic fever, Portugal, 2024; yellow triangle, the seropositive cases detected in the Beja district, Portugal, 1985. Blue numbered icons indicate the number of human cases reported in provinces in Spain since 2013 (Salamanca, 7; León, 3; Ávila, 2; Badajoz, 1; Cáceres, 1; Córdoba, 1; Madrid, 1; and Toledo, 1).

**Figure 2:**
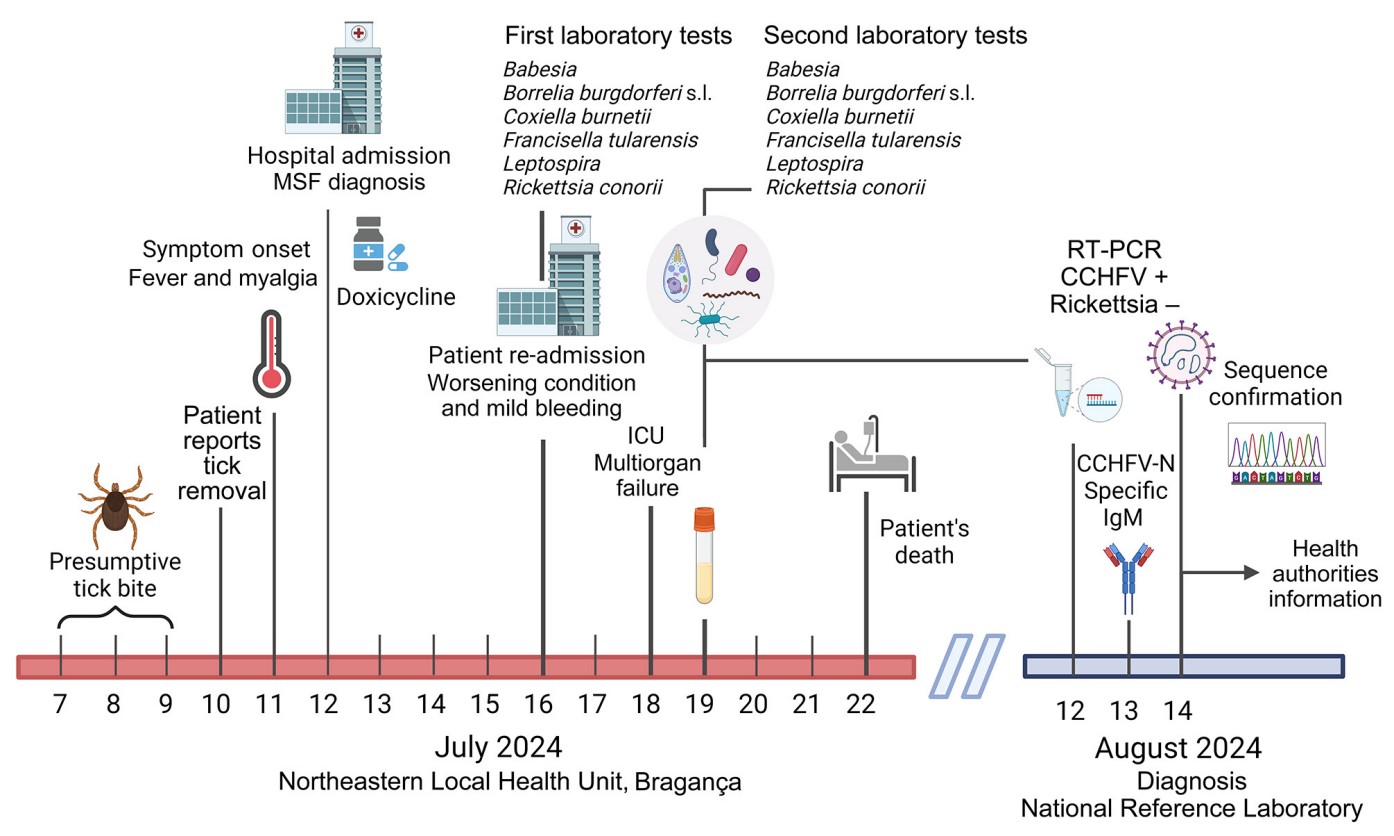
Timeline for fatal case of Crimean-Congo hemorrhagic fever, Portugal, 2024. Created using BioRender.com. CCHFV, Crimean-Congo hemorrhagic fever virus; ICU, intensive care unit; MSF, Mediterranean spotted fever; N, nucleoprotein; s.l., sensu lato; +, positive; –, negative.

On July 12, 2024, an 83-year-old man was admitted to Bragança Public Hospital (Bragança, Portugal) with complaints of fever (39.5°C) and myalgia. The patient reported removing a tick on July 10 (Table 1) from the periumbilical region 1 day before the onset of symptoms on July 11. He lived in the district of Bragança, in a rural environment, and mentioned participation in outdoor social events and activities from June 29–July 7; he had no history of international travel (Figure 1). We made a presumptive diagnosis of Mediterranean spotted fever (MSF), and initiated treatment with doxycycline (200 mg/d) before discharge. However, on July 16, day 6 of symptoms, the patient was readmitted with gastrointestinal symptoms (nausea, vomiting, diarrhea) and persistent myalgia and fever. On physical examination, his temperature was 38.5°C, heart rate 125 beats/min, respiratory rate 25 breaths/min, and blood pressure 90/56 mm Hg; and he had a small periumbilical eschar and a petechial rash on his legs. Laboratory testing revealed thrombocytopenia, prolonged activated partial thromboplastin time, hypofibrinogenemia, elevated transaminases, lymphopenia, and hepatocellular cytolysis (Table 2). His condition gradually worsened, and gingival bleeding and epistaxis required transfusion. We treated the patient symptomatically: correction of hypovolemia and electrolyte imbalance caused by gastrointestinal disorders, correction of hemostasis (platelet transfusion and fibrinogen), and antimicrobial drug administration. On July 18, he was transferred to the intensive care unit because of multiple organ failure. We collected a second serum specimen on July 19 that tested negative for various tickborne pathogens. However, the specimen was positive for IgG against *Rickettsia* by using *Rickettsia conorii* IFA Slide (BIOCELL Diagnostics, https://biocelldx.com), which indicated past exposure to a spotted fever group *Rickettsia*. The patient died on July 22 (Figure 2).

**Table 1:** Epidemiologic and clinical data for patient in a fatal case of Crimean-Congo hemorrhagic fever, Portugal, 2024*

Category	Description
Patient information and event timeline	
Risk factors	Subsistence farmer; animal contact (chicken and donkey); living in a rural area
Comorbidity	Hypertension, hypercholesterolemia, and prostate hypertrophy
Event	
Epidemiologic exposure	Patient reports tick removal on Jul 10
Symptoms onset	Fever and myalgia, Jul 11
Presumptive diagnosis	Mediterranean spotted fever, Jul 12
Signs and symptoms	Fever, myalgia, nausea, vomit, and diarrhea, Jul 16
	Petechiae, mild epistaxis, and gingival bleeding, Jul 18
Test results	
Imaging, Jul 18	Pleural effusion, lung widespread infiltrates, ascites, no hepatomegaly, or splenomegaly
Serum sample data, Jul 19	
Real-time PCR	
CCHFV commercial	Ct 21.2
CCHFV in-house	Ct 28.7
* Rickettsia* spp.	Negative
Serology†	
CCHFV-GPC IgM	Negative (<16)
CCHFV-N IgM	Pos itive(128)
CCHFV-GPC IgG	Negative (<32)
CCHFV-N IgG	Negative (<32)
SFGR IgM	Negative (<32)
SFGR *Rickettsia* IgG	Positive (128)

*CCHFV, Crimean-Congo hemorrhagic fever virus; Ct, cycle threshold; GPC, glycoprotein; N, nucleoprotein; SFGR, spotted fever group *Rickettsia*. †Cutoff values: CCHFV IgM>16, CCHFV IgG>32, *Rickettsia* IgM>32, *Rickettsia* IgG>128.

**Table 2:** Laboratory data for patient in fatal case of Crimean-Congo hemorrhagic fever, Portugal, 2024*

Category (reference limit range)	D2	D6	D7	D8	D9	D10	D11
Hemoglobin, g/dL (14.0–17.5)	12.9	11.6	10.5	11.6	10.6	9.4	8.8
Leukocytes, 10^9^ cells/L (4.4–11.3)	7.83	5.95	4.50	4.83	3.64	2.94	2.36
Neutrophils, 10^9^ cells/L	7.44	4.22	3.11	3.10	2.82	2.29	1.82
Lymphocytes, 10^9^ cells/L	0.24	1.49	1.22	1.56	0.71	0.57	0.50
Platelet count, 10^9^/L (150–450)	156	6	5	7	13	14	10
C-reactive protein, mg/dL	0.15	3.44	1.95	1.57	0.75	0.66	1.03
Prothrombin time, s (9.4–12.5)	11.5	11.2	11	10.9	12.7	14.1	15.1
Activated partial thromboplastin time, s (25.1–36.5)	23.6	53.9	52.7	56.6	48.0	44.3	47.7
Fibrinogen, mg/dL	275	NT	NT	125	135	129	NT
Aspartate aminotransferase, IU/L (<35)	31	545	549	1,259	7,547	7,644	8,330
Alanine aminotransferase,e IU/L (<45)	19	141	149	370	2,272	2,471	2,659
Total bilirubin, mg/dL (0.3–1.2)	NT	0.81	0.93	1.49	2.57	3.92	5.34
Creatine kinase, IU/L (<171)	NT	372	NT	263	262	246	397
Lactate dehydrogenase, IU/L (<248)	285	2,278	1,879	2,495	8,655	9,712	10,980
Urine cultures	NT	Neg	NT	Neg	NT	NT	NT
Blood cultures	Neg	Neg	NT	Neg	NT	NT	NT

*CCHFV, Crimean-Congo hemorrhagic fever virus; D, day after symptom onset; Neg, negative; NT, not tested; Pos, positive.

The case was reviewed at a regular hospital medical meeting because of the unclear cause of death. Because of the recent CCHF cases in the neighboring regions in Spain, a day 9 serum sample from the patient was sent to the reference laboratory of the Portuguese National Institute of Health for CCHFV testing and PCR for *Rickettsi*a spp. by using the Genesig PCR Kit (Primerdesign Ltd, https://www.genesig.com). The sample tested positive for CCHFV. We inactivated the sample in the Biosafety Level 3 facility by using the QIAamp Viral RNA Kit (QIAGEN, https://www.qiagen.com), followed by extraction under Biosafety Level 2 conditions. We conducted molecular testing by using the RealStar CCHFV RT-PCR Kit 1.0 (Altona Diagnostics, https://altona-diagnostics.com) and confirmed by using an in-house real-time reverse transcription PCR (RT-PCR) protocol (*12*). We purified a fragment (122 bp) from the small segment that was amplified by conventional RT-PCR by using the primers described previously (*12*) and conducted Sanger sequencing (GenBank accession no. PQ200212). Similarity searches within the GenBank dataset by using the BLAST algorithm (*13*) showed 100% similarity to CCHFV genotype III strains circulating in Spain (2014 and 2016) and Mauritania (1984). We tested for the presence of specific antibodies against CCHFV by using the Crimean-Congo fever virus Mosaic 2 immunofluorescence assay (EUROIMMUN, https://www.euroimmun.com) for IgM and IgG against the glycoprotein (GPC–glycoprotein precursor) and nucleoprotein antigens. The patient’s serum was positive for the IgM nucleoprotein-specific antigen at 128 titers (cutoff for positivity >16) (Figure 2).

We describe an autochthonous case of CCHF in Portugal in a deceased patient infected by a tick bite. The patient resided in Bragança, a district with a high incidence of MSF, and was initially misdiagnosed as having MSF because of overlapping epidemiologic, clinical, and laboratory features with CCHF (*2*,*14*). At admission on July 11, the patient showed no signs of severe illness. Of consequence, doxycycline, the standard antimicrobial for MSF, was administered, and the patient was then discharged. Of note, the patient had not traveled outside the district in the 2 weeks before symptom onset. Although CCHFV circulation has long been recognized in Portugal (*4*) and routine surveillance by RT-PCR is performed on *Hyalomma* spp. ticks collected throughout the country (*5*), the virus had not previously been detected by RT-PCR. All risk factors considered for CCHF infection in Portugal are similar to those known in Spain, including a favorable climate, the widespread presence of a tick vector, the country's geographic location along the migratory route of birds from CCHFV endemic areas, and proximity to Africa (*15*).

## Conclusions

This case highlights the public health threat posed by CCHFV in Portugal, particularly because of the widespread distribution of *Hyalomma* ticks in the country. Increased vector surveillance and studies with more tick samples from ungulates, especially red deer, as well as serosurveys in wildlife and humans are urgently needed. Public awareness campaigns should focus on preventative behaviors for avoiding tick exposure, especially among outdoor enthusiasts and professionals in nature-related activities.

Although attention to CCHF is most often drawn by severe cases, the clinical manifestations might be mild and could go unnoticed by clinicians. This case emphasizes the importance of including CCHF in the differential diagnosis list of MSF. Because of the high biologic risk of CCHFV transmission and the biosafety conditions required for its handling, prompt risk assessment is critical for rapid diagnosis of suspected cases.
